# How high is the level of technical noise in microarray data?

**DOI:** 10.1186/1745-6150-2-9

**Published:** 2007-04-11

**Authors:** Lev Klebanov, Andrei Yakovlev

**Affiliations:** 1 Department of Probability and Statistics, Charles University, Sokolovska 83, Praha-8, CZ-18675, Czech Republic; 2 Department of Biostatistics and Computational Biology, University of Rochester, 601, Elmwood Avenue, Box 630, Rochester, New York 14642, USA

## Abstract

**Background:**

Microarray gene expression data are commonly perceived as being extremely noisy because of many imperfections inherent in the current technology. A recent study conducted by the MicroArray Quality Control (MAQC) Consortium and published in *Nature Biotechnology *provides a unique opportunity to probe into the true level of technical noise in such data.

**Results:**

In the present report, the MAQC study is reanalyzed in order to quantitatively assess measurement errors inherent in high-density oligonucleotide array technology (Affymetrix platform). The level of noise is directly estimated from technical replicates of gene expression measurements in the absence of biological variability. For each probe set, the magnitude of random fluctuations across technical replicates is characterized by the standard deviation of the corresponding log-expression signal. The resultant standard deviations appear to be uniformly small and symmetrically distributed across probe sets. The observed noise level does not cause any tangible bias in estimated pair-wise correlation coefficients, the latter being particularly prone to its presence in microarray data.

**Conclusion:**

The reported analysis strongly suggests that, contrary to popular belief, the random fluctuations of gene expression signals caused by technical noise are quite low and the effect of such fluctuations on the results of statistical inference from Affymetrix GeneChip microarray data is negligibly small.

**Reviewers:**

The paper was reviewed by A. Mushegian, K. Jordan, and E. Koonin.

## 1. Background

It is widely believed that a high level of technical noise in microarray data is the most critical deterrent to the successful use of this technology in studies of normal and abnormal biological processes. In particular, the notorious lack of reproducibility of lists of detected genes across platforms and laboratories, as well as validation problems associated with prognostic signatures, is frequently attributed to this "flaw" of microarray technology [1,2]. This common belief also serves as a motivation for risky normalization procedures. Strange as it may seem, it was not until recently that a specially designed metrological study was reported by the MicroArray Quality Control (MAQC) Consortium in *Nature Biotechnology *[3]. This long overdue study, led by FDA scientists, is still far from being comprehensive but it provides valuable information for the technological noise assessment. Presented below are the results of our analysis of a subset of Affymetrix GeneChip data included in the MAQC data set. These results lead us to conclude that the variability in microarray data caused by technical noise is low and its role in statistical methodology of data analysis, exemplified by estimation of correlation coefficients, is far from critical.

## 2. Results and Discussion

To formulate the problem under consideration, let *x*_*ij*_, *i *= 1,..., *m*, *j *= 1,..., *n*, be the true log-expression of the *i*th gene on the *j*th array, where *m *is the total number of genes (probe sets) and *n *is the number of arrays (sample size). The unobservable random signals *x*_*ij *_are expected to be highly variable due to the inherent biological (inter-subject) variability. The most widely accepted array-specific random effect model of a technical noise in microarray data is given by

xij∗
 MathType@MTEF@5@5@+=feaafiart1ev1aaatCvAUfKttLearuWrP9MDH5MBPbIqV92AaeXatLxBI9gBaebbnrfifHhDYfgasaacH8akY=wiFfYdH8Gipec8Eeeu0xXdbba9frFj0=OqFfea0dXdd9vqai=hGuQ8kuc9pgc9s8qqaq=dirpe0xb9q8qiLsFr0=vr0=vr0dc8meaabaqaciaacaGaaeqabaqabeGadaaakeaacqWG4baEdaqhaaWcbaGaemyAaKMaemOAaOgabaGaey4fIOcaaaaa@31F9@ = *x*_*ij *_+ *ε*_*j*_,

where xij∗
 MathType@MTEF@5@5@+=feaafiart1ev1aaatCvAUfKttLearuWrP9MDH5MBPbIqV92AaeXatLxBI9gBaebbnrfifHhDYfgasaacH8akY=wiFfYdH8Gipec8Eeeu0xXdbba9frFj0=OqFfea0dXdd9vqai=hGuQ8kuc9pgc9s8qqaq=dirpe0xb9q8qiLsFr0=vr0=vr0dc8meaabaqaciaacaGaaeqabaqabeGadaaakeaacqWG4baEdaqhaaWcbaGaemyAaKMaemOAaOgabaGaey4fIOcaaaaa@31F9@ is the observable log-expression level and *ε*_*j *_is a log-additive random measurement error (noise). For each *i*, the random variables *x*_*ij *_and *ε*_*j *_are assumed to be independent. This model is clearly unidentifiable as no inference of the noise component *ε*_*j *_is possible from the observed signal xij∗
 MathType@MTEF@5@5@+=feaafiart1ev1aaatCvAUfKttLearuWrP9MDH5MBPbIqV92AaeXatLxBI9gBaebbnrfifHhDYfgasaacH8akY=wiFfYdH8Gipec8Eeeu0xXdbba9frFj0=OqFfea0dXdd9vqai=hGuQ8kuc9pgc9s8qqaq=dirpe0xb9q8qiLsFr0=vr0=vr0dc8meaabaqaciaacaGaaeqabaqabeGadaaakeaacqWG4baEdaqhaaWcbaGaemyAaKMaemOAaOgabaGaey4fIOcaaaaa@31F9@ without additional restrictive assumptions. This explains why no reliable estimates have been reported in the literature to date. The only scientifically sound way around this obstacle is to eliminate random fluctuations in the true biological signals *x*_*ij *_in specially designed experiments by producing replicate measurements on one and the same biological sample. This is exactly the design that was used in the MAQC study for each test-site and microarray platform. The MAQC data are publicly available via several websites [3].

In the present paper, we limit ourselves to the Affymetrix GeneChip platform referring to different test-sites (labs) as they are numbered in the database. There were six test-sites in the MAQC study, each repeatedly assaying four RNA pools to produce five technical replicates per pool. Two pools (UHRR and UBRR) consisted of pure RNA sample types while the other two were mixtures of these original samples. We limited our analysis to 100% UHRR (Sample A) and 100% UBRR (Sample B), because their mixtures are of limited utility in noise assessment. The reason for this statement is that mixing two samples with separated means may induce spurious variability in the data which has nothing to do with the technical noise we want to estimate. In what follows, we report only the results for Sample A as the results for Sample B are similar.

We used the Bioconductor RMA procedure to extract expression signals from perfect match probes in the original CEL files. As expected, the RMA background correction procedure induced an additional variability in the noise component *ε*_*j*_. The cause for this effect is obvious for those correction procedures that subtract one random variable from another while these variables tend to be positively correlated. Model-based methods for background correction resort to pooling across features in an effort to account for the background noise. This pooling of heavily dependent variables can make the resultant expression measures highly unstable. A similar effect was documented at the probe-set level [4,5]. Therefore, the reported results were obtained without background correction. It should be noted that the effect of background correction is quite weak and cannot affect the main conclusion drawn from our analysis.

Under the MAQC study design, model (1) assumes the form:

xij∗
 MathType@MTEF@5@5@+=feaafiart1ev1aaatCvAUfKttLearuWrP9MDH5MBPbIqV92AaeXatLxBI9gBaebbnrfifHhDYfgasaacH8akY=wiFfYdH8Gipec8Eeeu0xXdbba9frFj0=OqFfea0dXdd9vqai=hGuQ8kuc9pgc9s8qqaq=dirpe0xb9q8qiLsFr0=vr0=vr0dc8meaabaqaciaacaGaaeqabaqabeGadaaakeaacqWG4baEdaqhaaWcbaGaemyAaKMaemOAaOgabaGaey4fIOcaaaaa@31F9@ = *c*_*i *_+ *ε*_*j*_,

where *c*_*i *_is a gene-specific (non-random) constant. Since no gold standard has been established so far to tune the technological process at every test-site, the systematic error in xij∗
 MathType@MTEF@5@5@+=feaafiart1ev1aaatCvAUfKttLearuWrP9MDH5MBPbIqV92AaeXatLxBI9gBaebbnrfifHhDYfgasaacH8akY=wiFfYdH8Gipec8Eeeu0xXdbba9frFj0=OqFfea0dXdd9vqai=hGuQ8kuc9pgc9s8qqaq=dirpe0xb9q8qiLsFr0=vr0=vr0dc8meaabaqaciaacaGaaeqabaqabeGadaaakeaacqWG4baEdaqhaaWcbaGaemyAaKMaemOAaOgabaGaey4fIOcaaaaa@31F9@ will inevitably vary from site to site, an expectation confirmed by our analysis. Developing such a standard is an urgent need as the use of spiked-in probes does not do a good job in this regard. The above line of reasoning suggests that the variance of observed signal, rather than its variation coefficient [3], is all that is important in assessing the level of technical noise in microarray data. We pointed out this aspect of the problem in our recent discussion of the MAQC study [6]. The estimated (across arrays) standard deviations of expression levels for each gene can be summarized by reporting their average across genes for each of the six test-sites. We denote the resultant estimates by σ^
 MathType@MTEF@5@5@+=feaafiart1ev1aaatCvAUfKttLearuWrP9MDH5MBPbIqV92AaeXatLxBI9gBaebbnrfifHhDYfgasaacH8akY=wiFfYdH8Gipec8Eeeu0xXdbba9frFj0=OqFfea0dXdd9vqai=hGuQ8kuc9pgc9s8qqaq=dirpe0xb9q8qiLsFr0=vr0=vr0dc8meaabaqaciaacaGaaeqabaqabeGadaaakeaaiiGacuWFdpWCgaqcaaaa@2E86@_*k*_, *k *= 1,..., 6. The following estimates were obtained: σ^
 MathType@MTEF@5@5@+=feaafiart1ev1aaatCvAUfKttLearuWrP9MDH5MBPbIqV92AaeXatLxBI9gBaebbnrfifHhDYfgasaacH8akY=wiFfYdH8Gipec8Eeeu0xXdbba9frFj0=OqFfea0dXdd9vqai=hGuQ8kuc9pgc9s8qqaq=dirpe0xb9q8qiLsFr0=vr0=vr0dc8meaabaqaciaacaGaaeqabaqabeGadaaakeaaiiGacuWFdpWCgaqcaaaa@2E86@_1 _= 0.092, σ^
 MathType@MTEF@5@5@+=feaafiart1ev1aaatCvAUfKttLearuWrP9MDH5MBPbIqV92AaeXatLxBI9gBaebbnrfifHhDYfgasaacH8akY=wiFfYdH8Gipec8Eeeu0xXdbba9frFj0=OqFfea0dXdd9vqai=hGuQ8kuc9pgc9s8qqaq=dirpe0xb9q8qiLsFr0=vr0=vr0dc8meaabaqaciaacaGaaeqabaqabeGadaaakeaaiiGacuWFdpWCgaqcaaaa@2E86@_2 _= 0.091, σ^
 MathType@MTEF@5@5@+=feaafiart1ev1aaatCvAUfKttLearuWrP9MDH5MBPbIqV92AaeXatLxBI9gBaebbnrfifHhDYfgasaacH8akY=wiFfYdH8Gipec8Eeeu0xXdbba9frFj0=OqFfea0dXdd9vqai=hGuQ8kuc9pgc9s8qqaq=dirpe0xb9q8qiLsFr0=vr0=vr0dc8meaabaqaciaacaGaaeqabaqabeGadaaakeaaiiGacuWFdpWCgaqcaaaa@2E86@_3 _= 0.106, σ^
 MathType@MTEF@5@5@+=feaafiart1ev1aaatCvAUfKttLearuWrP9MDH5MBPbIqV92AaeXatLxBI9gBaebbnrfifHhDYfgasaacH8akY=wiFfYdH8Gipec8Eeeu0xXdbba9frFj0=OqFfea0dXdd9vqai=hGuQ8kuc9pgc9s8qqaq=dirpe0xb9q8qiLsFr0=vr0=vr0dc8meaabaqaciaacaGaaeqabaqabeGadaaakeaaiiGacuWFdpWCgaqcaaaa@2E86@_4 _= 0.666, σ^
 MathType@MTEF@5@5@+=feaafiart1ev1aaatCvAUfKttLearuWrP9MDH5MBPbIqV92AaeXatLxBI9gBaebbnrfifHhDYfgasaacH8akY=wiFfYdH8Gipec8Eeeu0xXdbba9frFj0=OqFfea0dXdd9vqai=hGuQ8kuc9pgc9s8qqaq=dirpe0xb9q8qiLsFr0=vr0=vr0dc8meaabaqaciaacaGaaeqabaqabeGadaaakeaaiiGacuWFdpWCgaqcaaaa@2E86@_5 _= 0.184, σ^
 MathType@MTEF@5@5@+=feaafiart1ev1aaatCvAUfKttLearuWrP9MDH5MBPbIqV92AaeXatLxBI9gBaebbnrfifHhDYfgasaacH8akY=wiFfYdH8Gipec8Eeeu0xXdbba9frFj0=OqFfea0dXdd9vqai=hGuQ8kuc9pgc9s8qqaq=dirpe0xb9q8qiLsFr0=vr0=vr0dc8meaabaqaciaacaGaaeqabaqabeGadaaakeaaiiGacuWFdpWCgaqcaaaa@2E86@_6 _= 0.056. The median values are: 0.091, 0.092, 0.101, 0.651, 0.186, 0.055, respectively, indicating that the standard deviations tend to be symmetrically distributed across genes. All these estimates are uniformly small except for Site 4, the latter being clearly an outlier.

The sample size (*n *= 5) for each site is small and it makes sense to pool at least those observations that do not pertain to the outlying Site 4. Since the mean log-expression values for a given gene vary from site to site, the data need to be centered to the respective sample means (across arrays). The reason for this site-specific centering is to make the pooled data identically distributed and remove a bias in the estimate of their variance. This procedure amounts to taking the arithmetic mean of the sample variances for individual sites as a pertinent estimator of the population variance of the technical noise under study. No other normalization procedure was applied in accordance with the primary goal of our study.

**Figure 1:**
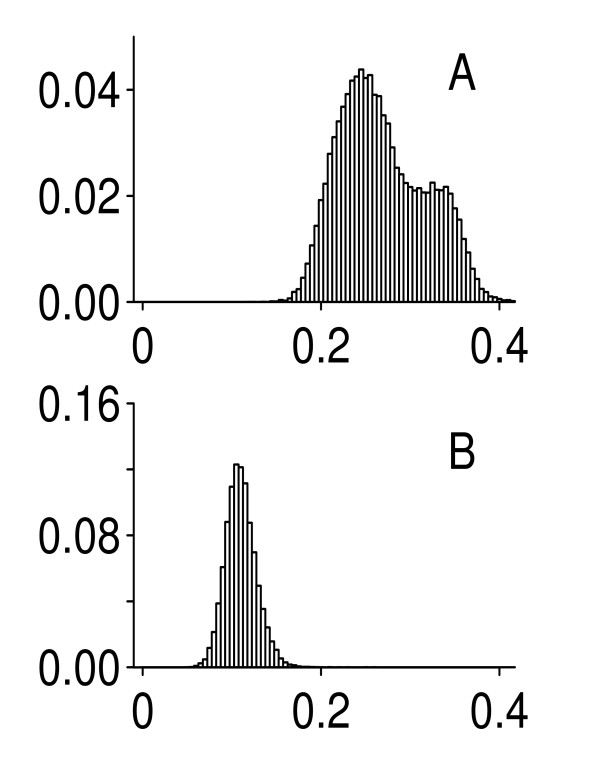
Histograms of standard deviations estimated from the MAQC data (original CEL files). A: pooled data for all test-sites; B: pooled data without Site 4.

Shown in Figure 1 are histograms of the σ^
 MathType@MTEF@5@5@+=feaafiart1ev1aaatCvAUfKttLearuWrP9MDH5MBPbIqV92AaeXatLxBI9gBaebbnrfifHhDYfgasaacH8akY=wiFfYdH8Gipec8Eeeu0xXdbba9frFj0=OqFfea0dXdd9vqai=hGuQ8kuc9pgc9s8qqaq=dirpe0xb9q8qiLsFr0=vr0=vr0dc8meaabaqaciaacaGaaeqabaqabeGadaaakeaaiiGacuWFdpWCgaqcaaaa@2E86@s pooled across genes and sites with (A) and without (B) the outlying Site 4. The second peak on the first histogram (Figure 1A) is due to Site 4. The overall mean value equals 0.267 in this case. The second histogram (Figure 1B) is unimodal with mean 0.108. In addition to having a small mean, this histogram is also quite narrow: the corresponding standard deviation is equal to 0.017 as compared to 0.049 for the first histogram. This observation is consistent with the multiplicative array-specific noise model given by formula (2). It should be emphasized that Site 4 is the only one (out of six sites!) where a high variability of expression signals is observed. Therefore, this is a site-specific problem and not a characteristic feature of the Affymetrix microarray technology in general.

The estimated average value of the standard deviation equaling 0.11 is quite small as compared to a value of 0.62 estimated from the data on patients (*n *= 88) with hyperdiploid (HYPERDIP) acute lymphoblastic leukemia. The patients were identified through the St. Jude Children's Research Hospital Database on childhood leukemia [7]. The discrepancy between the two estimates is attributable for the most part to the presence of biological variation in the HYPERDIP data. It is worth noting that the minimal (over the genes) value of standard deviations observed in the HYPERDIP data is equal to 0.37, a more than three-fold deviation from a value of 0.11 characterizing the average noise level.

**Figure 2:**
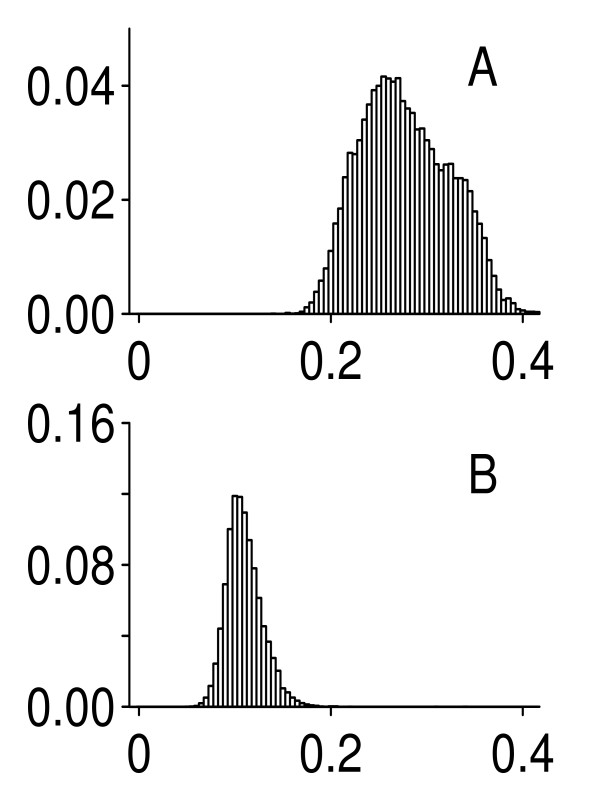
Histograms of standard deviations estimated from the MAQC data with updated probe set definitions. A: pooled data for all test-sites; B: pooled data without Site 4.

In response to one of the reviewers' concerns, the "gene versus probe set" issue was addressed to the best of our ability. Since the original probe set definitions in Affymetrix GeneChip data are known to be inaccurate [8], we updated them by using a custom CDF file to produce values of gene expressions. The CDF file was obtained from . This procedure is designed to remove multiple probe sets that map to single genes; it reduced the total number of probe sets from 54614 to 18027. The latter set is believed to be much more reliable in terms of gene identities. The HYPERDIP data were processed in the same way to result in 7084 probe sets. The results obtained with updated probe set definitions are shown in Figure 2. They are similar to those reported above. The average (across genes) of the standard deviations was equal to 0.107 (without Site 4), with all other characteristics remaining essentially the same. The corresponding overall mean for all sites was 0.275, a very small difference.

A natural question is: What effect may this small noise (with a standard deviation of 0.11 on the average) have on the results of statistical inference from microarray data? Since the correlation analysis [9], rather than two-sample tests, is particularly sensitive to the presence of technical noise, we addressed this issue in terms of the correlation coefficients in gene pairs. The coefficients were estimated from the HYPERDIP data and corrected for the technical noise using the following formula:

ρ(u,v)=ρ(u∗,v∗)(1+du,ε2)(1+dv,ε2)−du,εdv,ε
 MathType@MTEF@5@5@+=feaafiart1ev1aaatCvAUfKttLearuWrP9MDH5MBPbIqV92AaeXatLxBI9gBaebbnrfifHhDYfgasaacH8akY=wiFfYdH8Gipec8Eeeu0xXdbba9frFj0=OqFfea0dXdd9vqai=hGuQ8kuc9pgc9s8qqaq=dirpe0xb9q8qiLsFr0=vr0=vr0dc8meaabaqaciaacaGaaeqabaqabeGadaaakeaaiiGacqWFbpGCcqGGOaakcqWG1bqDcqGGSaalcqWG2bGDcqGGPaqkcqGH9aqpcqWFbpGCcqGGOaakcqWG1bqDdaahaaWcbeqaaiabgEHiQaaakiabcYcaSiabdAha2naaCaaaleqabaGaey4fIOcaaOGaeiykaKYaaOaaaeaacqGGOaakcqaIXaqmcqGHRaWkcqWGKbazdaqhaaWcbaGaemyDauNaeiilaWIae8xTdugabaGaeGOmaidaaOGaeiykaKIaeiikaGIaeGymaeJaey4kaSIaemizaq2aa0baaSqaaiabdAha2jabcYcaSiab=v7aLbqaaiabikdaYaaakiabcMcaPaWcbeaakiabgkHiTiabdsgaKnaaBaaaleaacqWG1bqDcqGGSaalcqWF1oqzaeqaaOGaemizaq2aaSbaaSqaaiabdAha2jabcYcaSiab=v7aLbqabaaaaa@5E5E@

where *ρ *(*u*, *v*) is the correlation coefficient between the true log-expression levels *u *and *v*, *ρ*(u*, v*) is its value in the presence of the noise *ε*, *d*_*u, ε *_= *σ*_*ε*_/*σ*_*u *_and *d*_*v*, *ε *_= *σ*_*ε*_/*σ*_*v *_are the noise/signal ratios, *σ*_*ε*_, *σ*_*u*_, and *σ*_*v *_are the corresponding standard deviations.

**Figure 3:**
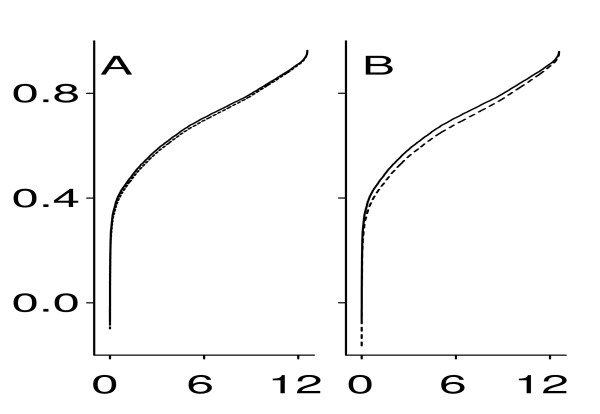
Correlation coefficients (in increasing order) estimated in all gene pairs formed by gene SLC25A24. *x*-axis: ordered pair number (× 1000), *y*-axis: correlation coefficient. Solid line: original correlation coefficients; dashed line: correlation coefficients corrected for a log-additive random noise with *σ*_*ε *_= 0.1 (A) and *σ*_*ε *_= 0.15 (B) in accordance with formula (3).

Formula (3) derives from model (1). Replacing all parameters in (3) with their empirical counterparts, one can estimate the true correlation *ρ*(*u*, *v*) from the noisy data (u*, v*) provided that *σ*_*ε *_is known. To estimate the unknown *σ*_*u*_, one can use the formula: Var(u*) = Var(*u*) + Var(*ε*). The same applies equally to *σ*_*v*_. Figure 3 presents a typical example of this analysis, displaying correlations in all pairs of genes formed by a given gene. The value of *σ*_*ε *_= 0.11 causes virtually no bias in the correlation coefficients (Figure 3A), and even the effect of a much higher level of noise (*σ*_*ε *_= 0.15) appears to be negligibly small (Figure 3B).

### Remark 1

How typical is the situation depicted in Figure 3A for all gene pairs formed by a single gene? To answer this question, we define the deviation of the corrected correlation coefficient ρ˜
 MathType@MTEF@5@5@+=feaafiart1ev1aaatCvAUfKttLearuWrP9MDH5MBPbIqV92AaeXatLxBI9gBaebbnrfifHhDYfgasaacH8akY=wiFfYdH8Gipec8Eeeu0xXdbba9frFj0=OqFfea0dXdd9vqai=hGuQ8kuc9pgc9s8qqaq=dirpe0xb9q8qiLsFr0=vr0=vr0dc8meaabaqaciaacaGaaeqabaqabeGadaaakeaaiiGacuWFbpGCgaacaaaa@2E82@ from its observed counterpart *ρ *as Δ = |ρ˜
 MathType@MTEF@5@5@+=feaafiart1ev1aaatCvAUfKttLearuWrP9MDH5MBPbIqV92AaeXatLxBI9gBaebbnrfifHhDYfgasaacH8akY=wiFfYdH8Gipec8Eeeu0xXdbba9frFj0=OqFfea0dXdd9vqai=hGuQ8kuc9pgc9s8qqaq=dirpe0xb9q8qiLsFr0=vr0=vr0dc8meaabaqaciaacaGaaeqabaqabeGadaaakeaaiiGacuWFbpGCgaacaaaa@2E82@ - *ρ*|. Computing Δs from microarray data and then taking their maximum is not a good idea because such an estimate is expected to have a large variance. However, a rough but more reliable estimate can be obtained by a numerical expedient. It follows from formula (3) that

Δ=|ρ˜−ρ|=|ρ{1−(1+du,ε2)(1+dv,ε2)}+du,εdv,ε|(1+du,ε2)(1+dv,ε2).
 MathType@MTEF@5@5@+=feaafiart1ev1aaatCvAUfKttLearuWrP9MDH5MBPbIqV92AaeXatLxBI9gBaebbnrfifHhDYfgasaacH8akY=wiFfYdH8Gipec8Eeeu0xXdbba9frFj0=OqFfea0dXdd9vqai=hGuQ8kuc9pgc9s8qqaq=dirpe0xb9q8qiLsFr0=vr0=vr0dc8meaabaqaciaacaGaaeqabaqabeGadaaakeaacqqHuoarcqGH9aqpdaabdaqaaGGaciqb=f8aYzaaiaGaeyOeI0Iae8xWdihacaGLhWUaayjcSdGaeyypa0ZaaSaaaeaacqGG8baFcqWFbpGCdaGadeqaaiabigdaXiabgkHiTmaakaaabaGaeiikaGIaeGymaeJaey4kaSIaemizaq2aa0baaSqaaiabdwha1jabcYcaSiab=v7aLbqaaiabikdaYaaakiabcMcaPiabcIcaOiabigdaXiabgUcaRiabdsgaKnaaDaaaleaacqWG2bGDcqGGSaalcqWF1oqzaeaacqaIYaGmaaGccqGGPaqkaSqabaaakiaawUhacaGL9baacqGHRaWkcqWGKbazdaWgaaWcbaGaemyDauNaeiilaWIae8xTdugabeaakiabdsgaKnaaBaaaleaacqWG2bGDcqGGSaalcqWF1oqzaeqaaOGaeiiFaWhabaWaaOaaaeaacqGGOaakcqaIXaqmcqGHRaWkcqWGKbazdaqhaaWcbaGaemyDauNaeiilaWIae8xTdugabaGaeGOmaidaaOGaeiykaKIaeiikaGIaeGymaeJaey4kaSIaemizaq2aa0baaSqaaiabdAha2jabcYcaSiab=v7aLbqaaiabikdaYaaakiabcMcaPaWcbeaaaaGccqGGUaGlaaa@7572@

Setting *σ*_*ε *_at 0.11, we make *d*_*u, ε *_= *σ*_*ε*_/*σ*_*u *_and *d*_*v*, *ε *_= *σ*_*ε*_/*σ*_*v *_to be functions of *σ*_*u *_and *σ*_*v *_only. A rough upper bound Δ_max _for the deviation Δ can be derived by maximizing the right-hand side of (4) with respect to *ρ*, *σ*_*u*_, and *σ*_*v *_under certain constraints imposed on these parameters by actual microarray data. In particular, all standard deviations observed in the updated HYPERDIP data are in the range from 0.30 to 1.21. A similar range is observed in numerous other data sets. Therefore, we maximize the right-hand side of (4) given *ρ *> 0 (see Remark 2) and *σ*_*u*_, *σ*_*v *_∈ [0.30, 1.21]. A numerical maximization resulted in Δ_max _= 0.155, a very small number in view of the roughness of our estimate. Consider the following example. An average non-corrected correlation coefficient of 0.94 was observed when correlations were estimated in all gene pairs from a subset of 1000 genes selected from the HYPERDIP data. Using Δ_max _= 0.155 we can correct this value to obtain ρ˜
 MathType@MTEF@5@5@+=feaafiart1ev1aaatCvAUfKttLearuWrP9MDH5MBPbIqV92AaeXatLxBI9gBaebbnrfifHhDYfgasaacH8akY=wiFfYdH8Gipec8Eeeu0xXdbba9frFj0=OqFfea0dXdd9vqai=hGuQ8kuc9pgc9s8qqaq=dirpe0xb9q8qiLsFr0=vr0=vr0dc8meaabaqaciaacaGaaeqabaqabeGadaaakeaaiiGacuWFbpGCgaacaaaa@2E82@ = 0.94 - 0.155 = 0.785 so that ρ˜
 MathType@MTEF@5@5@+=feaafiart1ev1aaatCvAUfKttLearuWrP9MDH5MBPbIqV92AaeXatLxBI9gBaebbnrfifHhDYfgasaacH8akY=wiFfYdH8Gipec8Eeeu0xXdbba9frFj0=OqFfea0dXdd9vqai=hGuQ8kuc9pgc9s8qqaq=dirpe0xb9q8qiLsFr0=vr0=vr0dc8meaabaqaciaacaGaaeqabaqabeGadaaakeaaiiGacuWFbpGCgaacaaaa@2E82@ > 0.7. This information can be used to provide a more accurate correction. Indeed, if we now maximize the right-hand side of (4) over *ρ *> 0.7 and *σ*_*u*_, *σ*_*v *_∈ [0.30, 1.21], the result will be Δ_max _= 0.043, thereby allowing us to state that ρ˜
 MathType@MTEF@5@5@+=feaafiart1ev1aaatCvAUfKttLearuWrP9MDH5MBPbIqV92AaeXatLxBI9gBaebbnrfifHhDYfgasaacH8akY=wiFfYdH8Gipec8Eeeu0xXdbba9frFj0=OqFfea0dXdd9vqai=hGuQ8kuc9pgc9s8qqaq=dirpe0xb9q8qiLsFr0=vr0=vr0dc8meaabaqaciaacaGaaeqabaqabeGadaaakeaaiiGacuWFbpGCgaacaaaa@2E82@ > 0.897.

The above calculations indicate that the presence of technical noise poses no serious problem for the statistical inference of correlation measures from microarray data, let alone two-sample comparisons. This is also an indirect indication that the effects of cross-hybridization are not of serious concern in modern Affymetrix data. Our rationale for the above statement is as follows. Since the competition of different oligonucleotide probes for the same transcript is random in nature, this process is expected to ultimately manifest itself in the observed technical variability, the latter having proven to be low. However, the proposed rationale is purely heuristic and cannot be independently verified as no technical vehicle is currently available for this purpose. The problem will probably become approachable after all "bad" probes are physically removed from the Affymetrix platform.

### Remark 2

Pairwise correlations between gene expression levels are known to be overwhelmingly positive [9-11]. In several data sets, we observed over 70% of all gene pairs to have their correlation coefficients greater than 0.75. See also Remark 1 regarding the average correlation coefficient observed in the HYPERDIP data. Normalization procedures tend to induce spurious negative correlations by interfering in the joint distribution of true biological signals and consequently in their correlation structure. This effect was documented in [9]. There are other adverse effects of normalization procedures for single-color arrays that cannot be ignored. For example, the distorting effect of quantile normalization manifests itself even in the marginal distributions of gene expression levels [12]. Our simulations (to be reported in another paper) have confirmed this conjecture showing that the quantile normalization procedure may dramatically reduce the variance of true expression signals. As a result, some null (not differentially expressed) genes may be selected by a two-sample test, thereby inducing an uncontrollable number of false discoveries. This is a high price for a gain in statistical power. It should be noted that the above-mentioned effect is not tangible with spiked-in probes because their intrinsic variability is low.

## 3. Conclusion

When gene expression microarray technology emerged some 10 years ago, there was a great deal of enthusiasm. A 1999 *Nature Genetics *paper [13] was entitled "Array of Hope". In the decade since the advent of microarray technology, there has accumulated a great deal of frustration among biologists who spend too much effort pursuing false leads and miss many important findings. As a reflection of this frustration, a 2005 *Nature Reviews *paper [14] was entitled "An Array of Problems". Numerous discussions in the literature show that there is a tendency to explain the notorious lack of power and instability of the results of data analysis by a high level of technical noise in the data [1,2]. At the same time, no attempt has been made to directly estimate this level for each gene in a situation where biological variability is absent. The MAQC study is the first one to offer such a possibility.

The above analysis of the MAQC data demonstrates that the magnitude of technical noise in microarray data has been gravely exaggerated, which point of view is likely to originate from the deterministic way of thinking that attributes all the variability in the data to measurement errors, thereby entirely ignoring the fact that the biological signal *x*_*ij *_is random. This latter statement should not be interpreted as a "chaotic behavior" of the biological signal, of course. The random nature of *x*_*ij *_has to do with biological variability; the latter is not a nuisance but a source of useful information. For example, the magnitude of inter-subject variability of gene expression is likely to reflect the tightness of control of a particular gene function by genomic regulatory mechanisms. Small sample sizes and inadequate statistical methodologies represent much more plausible explanations for many disturbing situations documented in the literature [6,15]. We agree with Dr. Mushegian that the level of noise in other microarray platforms have yet to be studied in a similar way. However, we would like to emphasize that the documented lack of reproducibility across platforms and laboratories cannot be attributed to a presumably high level of technical noise before other factors, such as small sample sizes and inadequate statistical methods, are ruled out.

There are many potential sources of technical variation in microarray data such as preparation of samples, labeling, hybridization, and other steps of microarray experiment. Such sources have been extensively discussed in the literature [16,17]. Our results show that, at least for the Affymetrix high-density oligonucleotide arrays, the contributions of these sources taken together result in a level of random noise which is deemed to be low for many practical purposes. This optimistic conclusion refers to modern-day technology, of course. However, studying such sources is very important in the context of systematic biases that represent the main obstacle to combining data from various laboratories. Recall that the systematic bias is characterized by the expected value of measurements and not by the standard deviation. This issue invites a special investigation and will hopefully lead to a universally accepted gold standard alluded to earlier in the present paper. We would like to emphasize that such an investigation calls for a special experimental design to eliminate biological variability similar to that in the MAQC study. The use of statistical methods for this purpose in the presence of biological variability is of little utility because the nonidentifiability aspect of underlying noise model will always stand in the way of such attempts. Pooling information across genes does not provide a way around this difficulty because the relevant statistical estimators constructed from pooled data cease to have the required properties [4,9].

The authors of the present report strongly believe that the potential of microarray technology is enormous. This resourceful technology yields abundant multivariate information on general quantitative regularities in the functioning of the whole genome machinery at the level of transcription. Such regularities have yet to be deciphered. Furthermore, the structure of other types of future high throughput biological data is envisioned to be quite similar (i.e. array-like) to that of gene expression microarrays. In particular, the basic statistical problems will remain the same even after *Solexa's *digital technology for gene expression profiling (see, ) has become widely available. We hope that the results of our analysis will positively affect the perception of usefulness of microarray technology. We also maintain the opinion [6] that the MAQC study should be continued to generate more technical replicates for a more accurate assessment of the random noise component.

## 4. Methods

The statistical methods employed in this paper are closely intertwined with the reported results and cannot be presented in a separate section. All the necessary technical details of data analysis and sources of data are given Section 2.

## Competing interests statement

The author(s) declare that they have no competing interests.

## Authors' contributions

Both authors contributed equally to this work.

## Reviewers' Comments

### Review 1 (Arcady Mushegian)

This is a most useful study that starts to set the record straight on the "problem" of technical noise in Affymetrix gene expression analysis platform. It is astonishing that such a study has not been performed, or at least not published, by the manufacturer or by anyone in the scientific community over more than a decade of utilizing GeneChips – until now.

My main question and concern is about the domain of applicability of the authors' conclusions. Here, some attention to careful wording could help. The main linguistic issue that remains to be sorted out is that the official name of the Affymetrix platform is "high-density oligonucleotide array", which in the past was sometimes contrasted with "microarrays", i.e., versions of the printed array technology. More recently, " microarray" has been casually broadened to signify any oligo-on-a-chip platform. I suppose that the authors need to declare what they are talking about, and what they are not, early and more often. For example, they say several times that their analysis concerns Affymetrix platform, and suggest at the very end that massive sequencing technologies, such as Solexa's, may have substantially similar statistical issues, but it is never stated whether or not anything in the current analysis is applicable to the ever-popular two-color arrays. Moreover, the statement in lines 3–5 of the Background (pg 2) is probably incorrect one way or the other, because low reproducibility "across platforms" may indeed be due, at least in part, due to high technical noise in some of said platforms. By the way, Lander's "Array of Hope" paper cited in the Conclusions deals mostly with printed two-color arrays, so citing it in this context is a bit misleading.

I suggest to change "all" to "many" in the last line of pg 7: the random noise in Affymetrix data may be high for some of such practical purposes as, for example, expression QTL analysis.

A disclaimer: I am not a statistician and cannot evaluate the equation (4).

### Review 2 (King Jordan)

The authors report a statistical re-analysis of the Affymetrix microarray data produced by the Microarray Quality Control (MAQC) Consortium in order to evaluate the level of technical noise in high-throughput gene expression data sets. They report that, one outlier data set notwithstanding, levels of technical replicate variation in gene expression levels measured by the Affymetrix platform are 'quite low'. This low technical variation (i.e. noise) does not lead to any 'serious problem' when measuring correlations between gene expression profiles. Accordingly, the authors insist that rumors of fatal flaws in microarray technologically have been greatly exaggerated.

In providing a review of this work, I would first like to issue a disclaimer: I am not a statistician and thus unqualified to evaluate the appropriateness (or lack thereof) of the statistical methods used in the paper. Having said that, the statistical models of technical noise in microarray data used in this paper are mercifully simple. Furthermore, the methods and results are presented clearly and succinctly. This combination of brevity and clarity is a real strength of the paper; the authors have evidently taken pains to ensure that the work is accessible to biologists for whom the implications are obviously most important. However, the lack of methodological and analytical detail is also a liability in some places. Fortunately, this can easily be remedied. Below, I detail some of the places where I believe that a greater depth of analysis and exposition would benefit the manuscript.

1. Expression level measure used. The authors re-analyze the MAQC data and use the original CEL files as a starting point. There are a number of different methods that can be used to extract 'expression levels' from the raw data in the CEL files, and it is not clear which method was used here. The Bioconductor RMA background correction procedure is mentioned in passing later in the manuscript, but it is not clear exactly how or whether this was employed to get the expression levels analyzed. More explicit detail should be provided here. In particular, it is important to know whether the authors used both perfect match and mismatch probes or perfect match only.

2. Gene versus probe (set) expression level. A more biologically pressing issue is the conflation of gene versus probe (set) in the manuscript. The authors state that they measure "correlation coefficients in gene pairs". However, there are many cases where multiple probe sets map to single genes. How were these handled? Are the correlation values discussed and shown only for probe set pairs?

4. Pairwise correlation analysis (Figure 3). The conclusion that pairwise gene (probe set?) correlations are not strongly affected by technical noise is an important one. While it seems to follow from the low technical noise illustrated here, the presentation of the results in Figure 3 could be far more convincing. In particular, the use of a single "typical example" raises a red flag. How typical is typical? Is this one of the examples with the lowest deviations between original and corrected correlation coefficients? Where does it fall in the distribution of all deviations? What does the greatest deviation look like? It would be far more useful to try and summarize all of the deviations observed as opposed to just presenting a single example. In addition, it isn't clear why all correlation values for any gene should be 0 or greater. Why are no negative values shown in Figure 3? For panel B, it would seem to be more germane to compare what happens to the corrected versus observed correlation coefficients in the presence of an average standard deviation of 0.267 (the level calculated before the removal of the outlier test site) instead of comparing to an arbitrarily higher noise level (0.15) as shown now.

5. Sources of variation. The sources of technical variation are not considered in this manuscript. Biologists will be very interested to know if there are any systematic and readily identifiable sources of technical noise in the Affymetrix probe sets and associated analytical tools used to derive expression levels. While the suggestions below regarding sources of variation are discretionary, a bit more work on this subject would strengthen the manuscript and make it even more useful to biologists.

5A. Sources of variation – expression level extraction. There are a number of analytical tools that are used to derive probe set expression levels from the raw data in CEL files. Among the most commonly used are MAS5, RMA and gc-RMA. As mentioned above, it is not entirely clear which method was used. In addition, it would be very interesting to know how the technical noise levels change when the different methods are used – e.g. which method gives the least noise.

5B. Sources of variation – probe GC content. The GC content of probes is can affect hybridization intensity (methods like gc-RMA correct for this). Is there any relationship between probe sequence context and the technical noise in the data analyzed here?

5C. Sources of variation – probe set count. Probe sets consist of different numbers (11–20 for newer arrays) of short oligonucleotides. Does the number of probes per set have any effect on the technical noise observed?

6. It is not clear how the low level of technical variation indicates that the effects of cross- hybridization are negligible (pg. 5). More evidence, or more explicit argument, needs to be offered to support this point. For instance, does the presence of closely related duplicate genes have any effect on technical noise levels?

7. A final minor point concerns the authors' contention that their conclusion of low technical noise in microarray data and the associated robustness of statistical analyses of these data represent a "radical change in the perception of the usefulness of microarray technology." This reads as an unnecessary overselling of their results. While many reservations about the reliability of microarray data have indeed been raised, there can also be little doubt that there is a broad consensus among biologists that these data are still very useful. The sheer number of microarray experiments, databases and publications supports this notion. They might consider toning this statement down a bit to emphasize that their results confirm the utility and reliability of this widely used technology.

### Review 3 (Eugene V. Koonin)

This paper addresses an obviously important problem and reports a potentially extremely important observation, namely, that the measurement error (technical noise) in Affymetrix microarray data is not particularly high and does not substantially affect statistical inferences made from microarray data, in particular, correlation coefficient estimates that are particularly sensitive to noise. This work is a rare case of a statistical study that come up with an optimistic message, namely, that the common perception of the extreme noisiness of expression array data making them useless for many purposes is a serious exaggeration.

I quite agree with the authors that this is a valuable message that needs to be brought to the attention of the large community of researchers using microarray results for their studies into diverse facets of biology. I am somewhat surprised that this simple analysis and straightforward conclusions were not part of the MAQC paper itself but, of course, such omissions are common enough. At the same time, I would like to note several issues that, in my opinion, require a somewhat more nuanced or more comprehensive approach.

First, the point of Klebanov's and Yakovlev's paper is not that the noise is low in some absolute sense but rather that it is low compared to a typical, biologically relevant signal. Put another way, when we detect a large difference between two expression profiles, it is unlikely to be due to technical noise and instead should be interpreted in biological terms. I believe this generally to be true; by the way, this case has been made in the literature, with comparisons to the differences between replicates used as an obvious standard – at the distinct risk of self-promotion, I will mention one of my own recent papers: Tsaparas et al. Global similarity and local divergence in human and mouse gene co-expression networks. BMC Evol Biol. 2006 Sep 12;6:70. However, it also has to be acknowledged that, for many situations, where subtle differences are involved, the level of technical noise will be a real problem. I cannot agree with the authors' statement (in the Conclusions) that the noise level is low for all practical purposes – there must be quite a few purposes, very practical ones, for which even this amount of noise will not be tolerable. In that respect, it does not help that the authors use only one real-life microarray study to showcase the lack of dramatic effect of technical noise. I suppose this will work as proof of principle but, perhaps, some more caution is needed in the conclusions.

Second, I suggest that the low level of technical noise reported by Klebanov and Yakovlev is the best case scenario. The replicates they analyze come from a specially launched, well-controlled study on microarray data quality. Indeed, even in that study, there was a serious outlier, test-site #4. I am afraid that, in real life, the noise level is, very often, greater; at least, this issues should be addressed.

Third, the authors suggest, in the Conclusions, that the popular notion of the prohibitive noisiness of microarray data stems from the deterministic view of biological data, in other words, disregard of intrinsic, biological noise (to me, this looks like better terminology than that used by the authors). I think this is a perceptive remark but it has to be made somewhat more clearly. I interpret Klebanov's and Yakovlev's statement that "the biological signal *x*_*ij *_is random in the sense that, due to the inevitable presence of intrinsic noise, this signal contains a random component and should be treated as a random variable. However, a reader could also take this to mean that the signal varies completely stochastically which is, of course, far from the truth. Furthermore, intrinsic noise in gene expression has been analyzed and discussed repeatedly, and some citations seem to be necessary here (e.g., Pedraza JM, van Oudenaarden A. Noise propagation in gene networks. Science 2005, 307: 1965–1969). In addition, I think that this deterministic worldview is not the only and, possibly, not even the principal source of the common perception of high technical noise in microarray data. Another and, possibly, more common reason is likely to be the genuinely high noise in early microarray experiments; I suspect that, in this case, technology progresses faster than many researchers realize.

Fourth, I think that the Background section is somewhat too succinct. Non-specialist readers would benefit from a few extra sentences explaining, in particular, in more quantitative terms, what were the previous estimates of the noise level.

In summary, I believe that this paper reports an important observation but could be improved by a more comprehensive discussion of the present and previous results, and perhaps, by slightly more cautious conclusions.
